# Dyadic Patterns of Patient and Caregiver Engagement in Type 2 Diabetes Mellitus Care: A Multicenter Observational Study

**DOI:** 10.1111/jocn.70186

**Published:** 2025-12-22

**Authors:** Diletta Fabrizi, Paola Rebora, Maria Grazia Valsecchi, Stefania Di Mauro, Michela Luciani, Davide Ausili

**Affiliations:** ^1^ School of Medicine and Surgery University of Milano‐Bicocca Monza Italy; ^2^ Bicocca Center of Bioinformatics, Biostatistics and Bioimaging (B4 Centre), School of Medicine and Surgery University of Milano‐Bicocca Monza Italy; ^3^ Biostatistics and Clinical Epidemiology, Fondazione IRCCS San Gerardo dei Tintori Monza Italy

**Keywords:** caregiver, chronic illness, glycated haemoglobin, latent class analysis, self care, type 2 diabetes mellitus

## Abstract

**Aim:**

To identify patterns of dyadic engagement in type 2 diabetes care, describe their characteristics, and explore their association with glycated haemoglobin.

**Background:**

In chronic conditions, patient self‐care and caregiver contribution should be considered a dyadic phenomenon. However, patterns of dyadic engagement in type 2 diabetes care have not yet been identified.

**Design:**

Multicentre observational cross‐sectional study.

**Methods:**

Patient self‐care and caregiver contribution were assessed using the Self‐Care of Diabetes Inventory and the Caregiver Contribution to Self‐Care of Diabetes Inventory. Patterns of dyadic engagement in type 2 diabetes care were identified by latent class analysis. Associations between patient‐caregiver characteristics and class membership were estimated using multinomial regression. The association between classes and glycated haemoglobin levels was assessed using linear regression.

**Results:**

251 dyads of patients with type 2 diabetes and their primary informal caregivers were enrolled. Patients were mostly male (55%, median age 72) and caregivers mostly female (71%, median age 64). Three patterns of dyadic engagement were identified: ‘equally engaged‐low care’ (14%), ‘mostly patient engaged‐middling care’ (25%), and ‘equally engaged‐high care’ (61%). Patient characteristics (sex, education, self‐efficacy) and caregiver characteristics (burden, chronic diseases) were associated with pattern membership. Membership in the ‘mostly patient engaged‐middling care’ and ‘equally engaged‐high care’ patterns was associated with decreased glycated haemoglobin compared to ‘equally engaged‐low care’.

**Conclusion:**

The three identified patterns of dyadic engagement in type 2 diabetes showed differences in patient and caregiver characteristics and were associated with glycated haemoglobin.

**Impact:**

The study identified and described patterns of dyadic engagement in type 2 diabetes care. The three identified patterns showed differences in characteristics and in patient glycemic control. Healthcare professionals should consider these patterns for tailoring interventions focused on both dyad members.

**Reporting Method:**

STROBE checklist was followed.

**Patient Contribution:**

Patients and their informal caregivers were recruited to participate in the study.

## What Does This Paper Contribute to the Wider Global Community?

- This study introduces the concept of patterns of dyadic engagement in type 2 diabetes care, supporting a more holistic approach to chronic disease management that includes both patients and their informal caregivers.
- By linking specific dyadic engagement patterns to glycemic outcomes, the study provides a practical framework for identifying dyads who may benefit from targeted support.
- These findings offer a foundation for developing tailored, dyad‐focused interventions that can enhance self‐care behaviours and improve clinical outcomes in diabetes care worldwide.

## 1 Introduction

It is estimated that currently 589 million adults aged 20–79 years are affected by diabetes, and this prevalence is expected to consistently increase in the coming years (International Diabetes Federation 2025). Over 90% of diabetes diagnoses correspond to type 2 diabetes mellitus (International Diabetes Federation 2025). Type 2 diabetes carries the weight of severe complications that contribute to elevated rates of morbidity, mortality and healthcare expenditures (Harding et al. 2019). However, patients' involvement in type 2 diabetes management had shown to be effective in mitigating the impact of these complications (American Diabetes Association Professional Practice Committee 2025a; International Diabetes Federation 2025).

## 2 Background

The process wherein individuals actively maintain their health and well‐being, recognise symptoms, and manage the challenges presented by illness is known as self‐care (Riegel et al. 2012, 2019). According to the Middle‐Range Theory of Self‐Care of Chronic Illness, the core concepts of self‐care are self‐care maintenance, self‐care monitoring, and self‐care management (Riegel et al. 2012, 2019). Self‐care maintenance comprises behaviours aimed at maintaining physical and emotional stability. Self‐care monitoring involves the vigilant observation of bodily signs and symptoms. Self‐care management entails the implementation of appropriate actions in response to such signs and symptoms (Riegel et al. 2012). In the context of diabetes, self‐care encompasses various activities, including adhering to dietary plans, following medication regimens, and adhering to physical activity recommendations (self‐care maintenance). It also involves the regular monitoring of blood glucose levels, body weight, foot health, and signs and symptoms of hypo‐ or hyperglycemia (self‐care monitoring), as well as the management of episodes characterised by hypo‐ or hyperglycemia (self‐care management) (Ausili et al. 2017; Riegel et al. 2012, 2019).

Although the key role of self‐care in improving glycemic control is widely recognised (American Diabetes Association Professional Practice Committee 2025a; Fabrizi et al. 2020; International Diabetes Federation 2025; Modarresi et al. 2020), patients may struggle in following recommendations of such complex and various behaviours (Bouldin et al. 2017; Riegel et al. 2012). Consequently, suboptimal glycemic control prevails among individuals living with type 2 diabetes (Afroz et al. 2019; Cedrick et al. 2021; Chetoui et al. 2020; Fiseha et al. 2018). To address this challenge, the presence of informal caregivers, such as family members or significant others, can be decisive in supporting patients in managing chronic conditions by facilitating the implementation of complex self‐care behaviours (Chen et al. 2017; Hooker et al. 2018). Caregiver contribution to patient self‐care entails providing assistance in managing the individual's health condition (Bouldin et al. 2017; Trivedi et al. 2012; Vellone et al. 2019, 2020), including assuming responsibility for tasks on the patient's behalf when independence is compromised (Pressler et al. 2013). In the context of chronic illnesses, caregiver contribution encompasses a range of crucial activities, such as emotional support, personal and clinical care (Buck et al. 2015; Clark et al. 2008), health promotion, symptoms monitoring, and shared decision‐making (Chen et al. 2017).

In investigating patient self‐care and caregiver contribution to it, a dyadic phenomenon should be recognised, wherein interdependence occurs between dyad members, as each one influences and is influenced by the other (Lyons and Lee 2018). Dyadic engagement in chronic condition management can be defined as the extent to which patients and caregivers jointly participate in and coordinate the behaviours required to manage the illness, reflecting their shared appraisal, collaboration and mutual influence in daily disease‐related activities (Lyons and Lee 2018). In the specific context of type 2 diabetes, a dyadic focus is particularly relevant due to the multifaceted nature of disease management, which includes monitoring blood glucose, adhering to complex medication regimens, maintaining dietary and physical activity routines, and preventing acute or long‐term complications (International Diabetes Federation 2025). Patients' self‐care is often influenced by caregiver support, including both practical assistance (e.g., meal planning, medication reminders) and motivational or emotional support (Chan et al. 2020). A dyadic perspective thus captures these interdependencies, providing a more comprehensive understanding of how the joint engagement of patients and caregivers can optimise illness management and potentially improve clinical outcomes (Vellone et al. 2019). Furthermore, the dyadic management of a chronic condition can be expressed according to a huge variability, contingent upon the stage of illness, the type of dyad, available support, and cultural factors (Lyons and Lee 2018). This heterogeneity represents a very useful asset for understanding the phenomenon of dyadic illness management, and it would therefore be essential to identify and describe it, albeit necessarily in a parsimonious way (Lee et al. 2020; Lyons and Lee 2018). To address this issue, distinct patterns of dyadic engagement in illness care have been identified in previous studies, involving patients with heart failure (Lee et al. 2015) or with multiple chronic conditions (De Maria et al. 2023) and their informal caregivers. To the best of our knowledge, a dyadic approach in assessing patient self‐care and caregiver contribution to it in type 2 diabetes has never been adopted. Furthermore, no previous studies have identified distinct patterns of dyadic engagement in type 2 diabetes care based on the combination of patient and caregiver involvement in behaviours specifically recommended for type 2 diabetes. Bringing this shortcoming could be a starting point to understand the dyadic functioning of type 2 diabetes management. Indeed, patterns identification could be useful to assess which patient and caregiver characteristics are associated with the tendency to implement a certain dyadic style of involvement in illness care. Furthermore, examining a potential association between dyadic pattern membership and critical health outcomes could help develop targeted interventions aimed at enhancing patient and caregiver engagement, which could subsequently lead to improved health outcomes.

## 3 The Study

The aims of this study were: (1) to identify patterns of dyadic engagement in type 2 diabetes care; (2) to identify factors associated with pattern membership at patient, caregiver and dyadic level; (3) to assess the association between patterns membership and patient glycemic control measured by glycated haemoglobin.

## 4 Methods

### 4.1 Design

A multicentre cross‐sectional study was conducted involving patients with type 2 diabetes and their informal caregivers from four outpatient diabetes clinics in the North of Italy between July 2018 and October 2019. This study was reported in accordance with the Strengthening the Reporting of Observational Studies in Epidemiology (STROBE) checklist.

### 4.2 Sample

During outpatient visits, a convenience sample of patient‐caregiver dyads was recruited. Patients were deemed eligible for inclusion if they fulfilled the diagnostic criteria for type 2 diabetes as outlined by guidelines (American Diabetes Association Professional Practice Committee 2025b), were aged 18 years or older, and provided written informed consent. Patients were excluded from the study if they had a recent (< 1 year from recruitment) type 2 diabetes diagnosis, were making their initial visit to the diabetes centre, showed difficulty in understanding the study questionnaire, or had confirmed cognitive impairment. Caregivers were eligible for participation if they were the primary informal caregiver for the patient, were aged 18 years or older, and provided written informed consent. Caregivers were excluded if they showed difficulties in understanding the study questionnaire or had confirmed cognitive impairment. Since this study addressed a pre‐specified secondary objective of a study primarily aimed at validating the Caregiver Contribution to Self‐Care of Diabetes Inventory, the sample size was estimated accordingly (Fabrizi et al. 2025). Given the aims of the present study, the available sample size was deemed acceptable, as it approached the level of approximately 300 observations generally recommended to ensure stable estimation and reliable class identification (Nylund‐Gibson and Masyn 2016; Weller et al. 2020).

### 4.3 Instruments

Sociodemographic data including age, biological sex, marital status, education level, employment status, and participation in educative sessions about diabetes were collected for both patients and caregivers. For caregivers, additional information was gathered, such as their relationship and cohabitation with the patient, daily hours of caregiving, years of caregiving, and the presence of at least one chronic disease. Clinical data from patients were obtained by reviewing medical records and included: time since type 2 diabetes diagnosis, unscheduled visits, hospitalisations, and emergency care access in the last year for any cause with their specified reason, diabetes complications, body mass index, number of comorbidities and glycated haemoglobin levels. The glycated haemoglobin level was considered in the present study as the main clinical outcome of dyadic type 2 diabetes management. Indeed, it is widely recognised as the gold standard for the assessment of the glycemic status in clinical practice and research, and it is strongly associated with diabetes‐related complications (American Diabetes Association Professional Practice Committee 2025c).

Patient self‐care and caregiver contribution to self‐care were measured respectively by the Self‐Care of Diabetes Inventory (Ausili et al. 2017) and the Caregiver Contribution to Self‐Care of Diabetes Inventory (Fabrizi et al. 2025), whose validity was already supported (Ausili et al. 2017; De Maria et al. 2022; Fabrizi et al. 2025). These two tools consist of the same three scales (self‐care/contribution to self‐care maintenance, monitoring, and management) assessing the same behaviours from the viewpoints of patients and caregivers, respectively. Specifically, the content of each single item is maintained, but the introduction is changed: where for patients the item starts with: ‘How often or routinely do you do these behaviours?’, for caregivers it is: ‘How often do you recommend the following behaviours to the person you care for? Or how often do you do these activities because the person you care for is not able to do them autonomously?’ The self‐care/contribution to self‐care maintenance scales assess behaviours to maintain physical and emotional stability (e.g., adherence to diet, medications, and physical activity recommendations). The self‐care/contribution to self‐care monitoring scales evaluate behaviours aimed at recognising signs and symptoms in the body (e.g., monitoring blood glucose, body weight, feet and symptoms of hypo‐ or hyperglycemia). The self‐care/contribution to self‐care management scales appraise behaviours employed in response to signs and symptoms (e.g., managing episodes of hypo‐ and hyperglycemia) (Ausili et al. 2017; Riegel et al. 2012). The Self‐Care of Diabetes Inventory and the Caregiver Contribution to Self‐Care of Diabetes Inventory also allowed to measure the patient self‐care self‐efficacy and the caregiver self‐efficacy in contributing to patient self‐care, respectively. With these scales, the patient confidence and persistence in self‐care behaviours or the caregiver confidence and persistence in supporting patient self‐care behaviours were investigated (Ausili et al. 2017; Fabrizi et al. 2025). Each item uses a 5‐point Likert‐type scale from ‘never’ to ‘always’, and each scale provides a 0–100 standardised score, where higher scores denote higher self‐care or higher contribution to self‐care (Ausili et al. 2017; Fabrizi et al. 2025). For the Self‐Care of Diabetes Inventory, the cut‐point used to classify self‐care as adequate or inadequate is ≥ 70 for self‐care maintenance and monitoring, and 60 for self‐care management (Fabrizi et al. 2020). Cut points have not yet been defined for the Caregiver Contribution to Self‐Care of Diabetes Inventory. The model‐based reliability index (omega) ranged from 0.81 to 0.89 for the Self‐Care of Diabetes Inventory (Ausili et al. 2017), and from 0.78 to 0.89 for the Caregiver Contribution to Self‐Care of Diabetes Inventory (Fabrizi et al. 2025), indicating good internal consistency for all scales. Both the instruments are available at https://self‐care‐measures.com/.

Diabetes‐related knowledge was measured by administering the Revised Brief Diabetes Knowledge Test, a valid and reliable self‐report tool that can be administered to both patients and caregivers (Baroni et al. 2023; Fitzgerald et al. 2016). The validation study of the Italian version of the Revised Brief Diabetes Knowledge Test was a previous publication from this study (Baroni et al. 2023). The Revised Brief Diabetes Knowledge Test consists of two sections, each one scored separately. The first section is composed of 14 items assessing general knowledge of diabetes regarding diet, glycemia, feet, physical activity, symptoms, and complications. The second section is an additional part of nine items to be completed only in the presence of insulin therapy, as this part investigates the knowledge about insulin therapy management. The score is the percentage of correct answers for each section (Baroni et al. 2023; Fitzgerald et al. 2016). The Italian version of the Revised Brief Diabetes Knowledge Test showed Kuder–Richardson Formula 20 values of 0.64 (general) and 0.73 (insulin‐specific) in patients, and 0.64 and 0.71, respectively, in caregivers, indicating acceptable reliability (Baroni et al. 2023).

Quality of life was measured by administering to both patient and caregiver the EuroQol‐five Dimensions Visual Analogue Scale, a 20‐cm vertical graded visual analogue scale having a value of 100 (i.e., the best possible health state) at its top and 0 (i.e., the worst possible health status) at its bottom (Rabin and De Charro 2001).

Mutuality was assessed by administering to both patient and caregiver the Mutuality Scale (Archbold et al. 1990; Pucciarelli et al. 2016). The Mutuality Scale is a 15‐item scale investigating four domains: love, shared pleasurable activities, shared values, and reciprocity. Each item is scored on a 5‐point Likert‐type scale from 0 (not at all) to 4 (a great deal). The total scale score consists of the mean of all item scores, ranging from 0 to 4. with higher scores meaning greater mutuality (Archbold et al. 1990; Pucciarelli et al. 2016). For the Italian version of the Mutuality Scale, the model‐based internal consistency index of the global score was 0.96 for both patients and caregivers.

Caregiver burden was measured by the Caregiver Burden Inventory. The Caregiver Burden Inventory is a self‐reported multidimensional tool that assesses caregiver burden, explored in terms of time‐dependence and developmental, physical, social, and emotional burden (Novak and Guest 1989). The Caregiver Burden Inventory comprises 24 items requiring a 5‐point Likert‐type scale response from 0 (minimum burden) to 4 (maximum burden). The Caregiver Burden Inventory provides a 0–100 total score or a 0–20 score for each subscale, where higher scores mean more burden (Greco et al. 2017; Novak and Guest 1989). The Italian version of the Caregiver Burden Inventory demonstrated high internal consistency reliability, with a Cronbach's alpha of 0.96 for the total score (Greco et al. 2017).

Caregiver preparedness was measured using the Caregiver Preparedness Scale, an eight‐item scale investigating the self‐perceived caregiver preparedness to care for patient physical and emotional needs, setting up services, coping with the stress of caregiving, making caregiving activities pleasant, responding and managing emergencies, getting help and information from the health care system, and overall preparedness. Each item is rated between 0 (not at all prepared) to 4 (very well prepared), and items are summed for a total score that can range from 0 to 32, with higher scores indicative of feeling better prepared for the caregiving role (Archbold et al. 1990; Pucciarelli et al. 2014). The Italian version of the Caregiver Preparedness Scale showed a composite reliability index of 0.93 and a Cronbach's alpha of 0.94 (Pucciarelli et al. 2014).

### 4.4 Data Analysis

To describe patients and caregivers, frequencies and percentages were used for categorical variables, median and interquartile range for continuous variables.

To identify distinct patterns of dyadic engagement in type 2 diabetes care, two steps were followed. First, multilevel unconditional models (i.e., with no predictors) were applied to each Self‐Care of Diabetes Inventory/Caregiver Contribution to Self‐Care of Diabetes Inventory scale, with the member of the dyad as a fixed effect and random intercept and slope terms to capture variability across dyads in average engagement levels and incongruence between dyad members. From these models, estimates of the dyadic average and incongruence in type 2 diabetes management were derived within each dyad and for each Self‐Care of Diabetes Inventory/Caregiver Contribution to Self‐Care of Diabetes Inventory scale (i.e., self‐care maintenance, self‐care monitoring and self‐care management) (De Maria et al. 2023; Lee et al. 2015; Lyons and Lee 2020). The dyadic average represented the mean of the dyadic involvement in the investigated behaviours among the two members of the dyad, while the dyadic incongruence reflected the magnitude and the direction of the incongruence in the dyadic involvement (negative values indicated that the caregiver contributed more than the patient and vice versa) (Lyons et al. 2002). Second, a latent class analysis was performed using the parameters estimated at the first step (six for each dyad: two predicted random effects for the three scales) (De Maria et al. 2023; Lee et al. 2015). The Bayesian Information Criteria (the lower the better), posterior probabilities (average posterior probabilities for most likely class near 1.0), the size of the observed profiles (not less than 5% of the sample), the model convergence (entropy near 1.0), the Lo–Mendell–Rubin adjusted likelihood ratio test, and the parametric bootstrap likelihood ratio test were used as fit indices (Lee et al. 2020; Ram and Grimm 2009). These indices were used to support the choice of the number of classes in the latent class analysis. Differences between identified patterns were tested using the Kruskal–Wallis test or the chi‐square test, as appropriate.

To identify factors associated with pattern membership, multinomial regression was adopted. Consistently with the Theory of Dyadic Illness Management (Lyons and Lee 2018) the following variables were initially theorised as potential determinants: age (patient, caregiver), biological sex (patient, caregiver), income (patient), occupational status (patient, caregiver), education level (patient, caregiver), years since diagnosis of type 2 diabetes (patient), presence of diabetes complications (patient), body mass index (patient), presence of insulin therapy (patient), hospitalisation, access to emergency care, or unscheduled visit in the last year (patient), education on diabetes (patient, caregiver), cohabitation (patient, caregiver), years of caregiving (caregiver), hours of caregiving per day (caregiver), presence of chronic disease (caregiver), as well as the scores in administered tools. In line with the aforementioned theory, which conceptualises illness management as a dyadic process influenced by both individual and shared characteristics of the patient and caregiver, sociodemographic, clinical, and caregiving‐related domains were prioritised, as they are expected to shape the level and quality of collaboration within the dyad (Lyons and Lee 2018). A backward stepwise procedure using the Akaike information criterion (Heinze et al. 2018) was applied on complete observations to select among the covariates. Age and biological sex of both patient and caregiver were included a priori. A complete case analysis was performed due to the limited number of observations containing missing data, which were assumed to follow a missing at random mechanism (Lachenbruch 2011; Ross et al. 2020). Results were reported as odds ratios (OR) and 95% confidence intervals (CI).

To investigate the association between pattern membership and patient glycemic control measured by glycated haemoglobin, linear regression was performed, adjusting for the following pre‐specified covariates: patient age, biological sex, body mass index, presence of diabetes complications, time since diagnosis of type 2 diabetes and presence of insulin therapy. These variables were selected a priori based on their well‐established associations with glycemic outcomes in type 2 diabetes (American Diabetes Association Professional Practice Committee 2025c) and their potential to confound the relationship between dyadic management patterns and glycemic control. In this model, the estimated glycated haemoglobin value was reported both as a percentage and in mmol/mol.

Statistical analyses were performed using Mplus (version 8.11) and R (version 4.4.2).

### 4.5 Ethical Considerations

The study received approval from the Institutional Review Board of each involved centre (Azienda Socio‐Sanitaria Territoriale Grande Ospedale Metropolitano Niguarda, Milan, Italy; Azienda Socio‐Sanitaria Territoriale Monza, Monza, Italy; Azienda Socio‐Sanitaria Territoriale Garda, Brescia, Italy; Azienda Socio‐Sanitaria Territoriale Lariana, Como, Italy), confirming that no further ethical approval was required. All enrolled participants provided signed informed consent. The study procedures adhered to the ethical standards set by the responsible committee on human experimentation, both at the institutional and national levels and followed the principles outlined in the Declaration of Helsinki and in the Declaration of Taipei.

## 5 Results

### 5.1 Characteristics of the Sample

A total of 251 patient‐caregiver dyads were enrolled. The proportion of missing values varied across variables, with the most affected having 6.7% missing data. Given the small proportion and random distribution of missing data, their potential impact on bias or generalizability is expected to be negligible. Patients were mostly male (55%, *n* = 138) aged 70 years or more (67%, *n* = 169), married or cohabiting with the partner or spouse (76%, *n* = 191), with a primary or secondary school education (76%, *n* = 190), and retired (79%, *n* = 198). Glycated haemoglobin value was over the goal of < 7% (53 mmol/mol) (American Diabetes Association Professional Practice Committee 2025c) for 59% of patients (*n* = 147), body mass index indicated overweight (≥ 25 kg/m^2^) for 78% of cases (*n* = 195), and more than half of patients had needed hospitalisation, emergency care access, or unscheduled visits in the last year (55%, *n* = 139). Caregivers were mostly female (71%, *n* = 178), aged 60 years or over (64%, *n* = 161), married or having a cohabitant partner (90%, *n* = 225), with a primary or secondary school education (59%, *n* = 148), and retired (55%, *n* = 138). Caregivers were mostly the spouse or partner of the patient (66%, *n* = 166) and were cohabitant with the patient (75%, *n* = 188). About a quarter of caregivers (25%, *n* = 63) spent at least 8 h/day on caregiving, and over 60% had been taking care of the patient for at least 10 years (63%, *n* = 157). Only 11% (*n* = 28) had received education on diabetes, and more than half of them stated that they had at least one chronic disease (55%, *n* = 139). Patients' and caregivers' characteristics are reported in Table 1.

**TABLE 1:** Patients' and caregivers' characteristics.

Characteristics	Patient	Caregiver
	*n* = 251	*n* = 251
	Median [1st−3rd quartile]	
Age (years)	72 [67–79]	64 [54–71]

	*n* (%)	
Biological sex		
Male	138 (55)	73 (29)
Marital status		
Married/Cohabitant	191 (76)	225 (90)
Divorced/Separated	7 (3)	13 (5)
Single/Never married	4 (2)	7 (3)
Widower/Widow	49 (19)	6 (2)
Education level		
Primary school	115 (46)	49 (20)
Secondary school	75 (30)	99 (39)
High school	51 (20)	93 (37)
University	10 (4)	10 (4)
Employment status		
Employed	26 (10)	78 (31)
Retired	198 (79)	138 (55)
Homemaker	9 (4)	13 (5)
Unemployed	17 (7)	22 (9)
Low income		
Yes	129 (51)	—
Relationship with patient		
Spouse/Partner	—	166 (66)
Son/Daughter		60 (24)
Brother/Sister		6 (2)
Son−/Daughter‐in‐law		7 (3)
Other		12 (5)
Cohabitation with patient		
Yes	—	188 (75)

	Median [1st–3rd quartile]	
Years since diagnosis	12 [6–20]	—
Hours of caregiving per day	—	3 [1–6]
Years of caregiving	—	10 [5–20]

	*n* (%)	
Presence of at least one chronic disease		
Yes	—	139 (55)
Education on diabetes		
Yes	89 (35)	28 (11)
Hospitalization^a^		
Yes	78 (31)	—
Access to emergency care^a^		
Yes	73 (29)	—
Unscheduled visit^a^		
Yes	41 (16)	—
Presence of diabetes complications		
Yes	102 (41)	—

	Median [1st–3rd quartile]	
Body mass index	28.1 [25.4–31.7]	—
HbA1c		—
(%)	7.1 [6.6–7.8]	
mmol/mol	54 [49–62]	
Number of comorbidities	3 [2–4]	—

*Note:* Missing data among patients: 1 in employment status; 1 in body mass index. Missing data among caregivers: 17 in hours of caregiving per day; 13 in years of caregiving.

^a^ In the last year.

Patients mostly reported adequate levels of self‐care maintenance (73%, *n* = 182 score ≥ 70), while self‐care monitoring (54%, *n* = 135 score < 70) and self‐care management (57%, *n* = 142 score < 60) were mostly inadequate. Furthermore, they mostly showed good self‐care self‐efficacy, answered just over half of the diabetes knowledge questions correctly, and reported good levels of quality of life and mutuality (Table 2). The majority of caregivers reported poor scores in Caregiver Contribution to Self‐Care of Diabetes Inventory scales. Furthermore, they mostly showed good levels of self‐efficacy, answered over half of the diabetes knowledge questions correctly, reported high quality of life and mutuality, low burden, and discrete preparedness (Table 2).

**TABLE 2:** Patients' and caregivers' scores in administered tools.

Tools	Patient	Caregiver
	*n* = 251	*n* = 251
	Median [1st−3rd quartile]	
Self‐care maintenance (SCODI, 0–100)	79 [69–88]	—
Self‐care monitoring (SCODI, 0–100)	68 [50–85]	—
Self‐care management (SCODI, 0–100)	56 [26–75]	—
CC to self‐care maintenance (CC‐SCODI, 0–100)	—	60 [38–75]
CC to self‐care monitoring (CC‐SCODI, 0–100)	—	56 [29–76]
CC to self‐care management (CC‐SCODI, 0–100)	—	53 [19–81]
Self‐care self‐efficacy (SCODI, 0–100)	75 [59–91]	—
Caregiver self‐efficacy in contributing to patient self‐care (CC‐SCODI, 0–100)	—	75 [52–90]
Diabetes knowledge – General (DKT2, 0–100)	57 [43–71]	64 [50–71]
Diabetes knowledge – Insulin^a^ (DKT2, 0–100)	56 [33–78]	56 [44–78]
Quality of life (EQ5D‐VAS, 0–100)	70 [50–80]	80 [70–90]
Mutuality (MS, 0–4)	3.2 [2.8–3.6]	3.1 [2.5–3.6]
Caregiver burden (CBI, 0–100)	—	7 [2–17]
Caregiver preparedness (CPS, 0–32)	—	19 [15–24]

*Note:* Missing data among patients: 1 in Mutuality. Missing data among caregivers: 8 in Diabetes knowledge—Insulin; 3 in Mutuality.

Abbreviations: CBI, Caregiver Burden Inventory; CC, caregiver contribution; CC‐SCODI, Caregiver Contribution to Self‐Care of Diabetes Inventory; CPS, Caregiver Preparedness Scale; DKT2, Revised Diabetes Knowledge Test; EQ5D‐VAS, EuroQol Five‐Dimension–Visual Analogue Scale; MS, Mutuality Scale; SCODI, Self‐Care of Diabetes Inventory.

^a^ Responses required only for patients with insulin therapy (*n* = 105) and their caregivers.

After estimating the dyadic average and incongruence for each dyad and for each self‐care/caregiver contribution dimension, we fitted latent class analyses assuming from 2 to 5 classes. Table S1 reports the related indices of fit.

**FIGURE 1:**
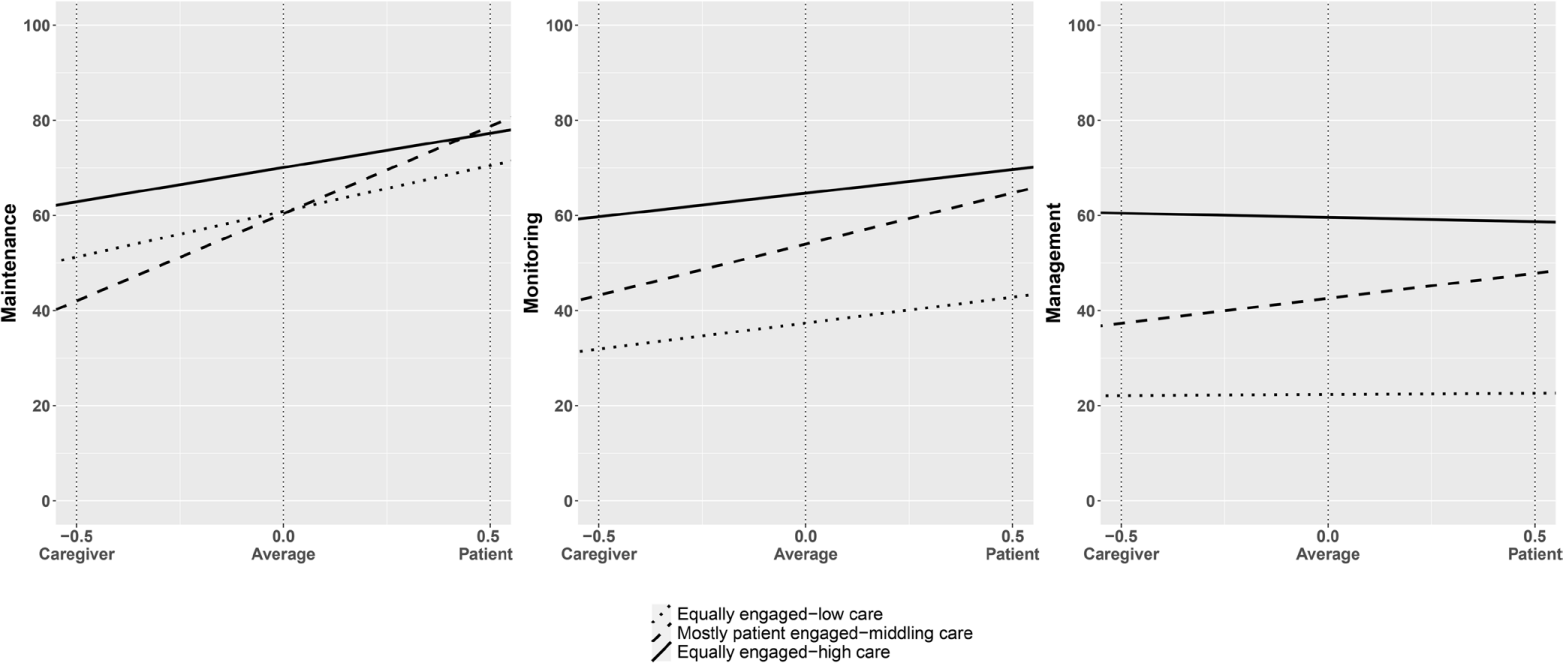
Mean dyadic engagement in type 2 diabetes care behaviours of the identified classes. The *x*‐axis reports the dyad member (−0.5 refers to the caregiver and 0.5 to the patient), while the *y*‐axis reports the self‐care maintenance, monitoring, or management behaviours' score. Thus, the level of self‐care (*y*‐axis) corresponding to: (i) −0.5 on the *x*‐axis coincides with the estimated mean of caregiver contribution to self‐care maintenance, monitoring, or management behaviours in each class; (ii) 0.0 on the *x*‐axis coincides with the estimated mean dyadic engagement in self‐care maintenance, monitoring, or management behaviours in each class; (iii) 0.5 on the *x*‐axis coincides with the estimated mean of patient self‐care maintenance, monitoring, or management behaviours in each class.

The model with three classes was the one that, in addition to displaying the highest entropy and good performances in further fit indices (entropy = 0.871; lowest classification probability = 0.910; Lo–Mendell–Rubin adjusted likelihood ratio test *p* = 0.0190; parametric bootstrap likelihood ratio test *p* < 0.001), identified better characterised and interpretable classes (Figure S1). Figure 1 reports the level of self‐care engagement for patients and caregivers in the three classes. Labels of the three classes were attributed consistently with their dyadic engagement in type 2 diabetes care (Figure 1 and Table S2).

The demographic and clinical characteristics of patients and caregivers in the identified classes are presented in Table 3.

**TABLE 3:** Characteristics of patients, caregivers and dyads in the three classes of dyadic engagement in type 2 diabetes mellitus care.

Characteristics	Equally engaged‐low care	Mostly patient engaged‐middling care	Equally engaged‐high care	*p*
	*n* = 34	*n* = 63	*n* = 154	
Patient				
Age (years)	72 [65–80]	72 [65–79]	73 [67–78]	0.775
Biological sex: Male	23 (68)	32 (51)	83 (54)	0.256
Marital status: Married/Cohabitant^a^	27 (79)	52 (82)	112 (73)	0.272
Education: High school/University^b^	5 (15)	26 (41)	30 (19)	0.001
Employment status: Employed^c^	3 (9)	9 (14)	14 (9)	0.505
Low income: Yes	14 (41)	33 (52)	82 (54)	0.437
Education on diabetes: Yes	15 (44)	18 (29)	56 (36)	0.095
Hospitalisation: Yes	17 (50)	19 (30)	42 (27)	0.034
Access to emergency care: Yes	11 (32)	19 (29)	44 (29)	0.903
Unscheduled visit: Yes	6 (18)	13 (21)	22 (14)	0.504
Diabetes complication(s): Yes	14 (41)	29 (46)	59 (38)	0.574
Body mass index (kg/m^2^)	28.1 [26.0–32.1]	26.9 [24.9–30.6]	28.4 [25.5–32.5]	0.248
Time since diagnosis (years)	9 [3–22]	12 [6–20]	12 [7–20]	0.264
Number of comorbidities	3 [2–4]	3 [2–4]	3 [2–4]	0.830
Self‐care self‐efficacy (SCODI)	74 [50–86]	80[64–100]	77 [64–91]	0.118
Diabetes knowledge – General (DKT2)	53 [38–71]	57 [43–71]	57 [43–64]	0.381
Diabetes knowledge – Insulin (DKT2)	55 [44–75]	67 [33–89]	56 [33–78]	0.439
Quality of life (EQ5D – VAS)	70 [66–80]	70 [60–80]	70 [50–80]	0.512
Mutuality (MS)	3.3 [2.8–3.6]	3.3 [2.8–3.8]	3.2 [2.8–3.6]	0.573
HbA1c (%)	7.3 [6.7–8.3]	7.0 [6.5–7.7]	7.2 [6.5–7.8]	0.212
HbA1c (mmol/mol)	56 [50–67]	53 [48–61]	55 [48–62]	
Caregiver				
Age	64 [55–74]	66 [54–71]	64 [53–71]	0.672
Biological sex: Male	10 (29)	17 (27)	46 (30)	0.913
Marital status: Married/Cohabitant^a^	29 (85)	58 (92)	138 (90)	0.580
Education: High school/University^b^	12 (35)	30 (47)	61 (40)	0.423
Employment status: Employed^c^	8 (23)	21 (33)	49 (32)	0.579
Education on diabetes: Yes	5 (15)	11 (18)	12 (8)	0.095
Chronic disease(s): Yes	11 (32)	37 (59)	91 (59)	0.015
Years of caregiving	7 [2–10]	11 [5–18]	10 [6–20]	0.065
Hours of caregiving per day	4 [2–8]	3 [2–7]	3 [1–4]	0.136
Self‐efficacy in contributing to patient self‐care (CC‐SCODI)	69 [50–92]	75 [56–100]	75 [52–86]	0.238
Diabetes knowledge – General (DKT2)	64 [50–71]	64 [50–71]	64 [50–71]	0.710
Diabetes knowledge—Insulin (DKT2)	67 [47–78]	67 [44–89]	56 [33–78]	0.305
Quality of life (EQ5D—VAS)	80 [75–90]	80 [70–90]	80 [66–90]	0.512
Mutuality (MS)	3.2 [2.9–3.6]	3.2 [2.6–3.7]	3.0 [2.5–3.5]	0.096
Burden (CBI)	4 [1–11]	7 [2–17]	7 [3–18]	0.222
Preparedness (CPS)	19 [13–24]	22 [16–24]	18 [15–24]	0.574
Dyad				
Cohabitation: Yes	25 (74)	46 (73)	117 (76)	0.509
Relationship type				
Spousal	23 (68)	42 (67)	101 (66)	0.276
Adult‐child	6 (18)	12 (19)	42 (27)	
Other family/friend	5 (15)	9 (14)	11 (7)	

*Note:* Differences between identified classes were tested using the Kruskal–Wallis test or the chi‐square (*χ* ^2^) test, as appropriate.

Abbreviations: CBI, Caregiver Burden Inventory (0–100); CC, caregiver contribution; CC‐SCODI, Caregiver Contribution to Self‐Care of Diabetes Inventory (0–100); CPS, Caregiver Preparedness Scale (0–32); DKT2, Revised Diabetes Knowledge Test (0–100); EQ5D‐VAS, EuroQol Five‐Dimension—Visual Analogue Scale (0–100); MS, Mutuality Scale (0–4); SCODI, Self‐Care of Diabetes Inventory (0–100).

^a^ Complementary category includes divorced/separated, single/never married, and widower/widow.

^b^ Complementary category includes primary school and secondary school.

^c^ Complementary category includes retired, homemaker, and unemployed.

First, the dyadic class labelled as ‘equally engaged‐low care’ (14%, *n* = 34) showed a low dyadic average in self‐care maintenance behaviours and the lowest dyadic averages in self‐care monitoring and management behaviours with slight incongruence between patient self‐care and caregiver contribution (Figure 1 and Table S2). In this dyadic class, patients had a median age of 72 years, were mostly retired male, with a primary or secondary school education and high income. Furthermore, their median time since diabetes diagnosis was 9 years and more than 40% of them had at least one diabetes complication. Caregivers belonging to this dyadic class had a median age of 64 years, were mostly retired female or homemakers with a primary or secondary school education. They had been engaged in caregiving for a median of 7 years and referred to spending a median of 4 h/day on caregiving activities. The vast majority of the dyads of this class were cohabitant and the most frequent type of relationship existing between the two members of the dyad is the spousal one. As compared to the other classes, patients belonging to this class had the lowest level of education and were the most hospitalised over the last year. Furthermore, caregivers had a chronic disease less often than the others (Table 3).

The second class labelled as ‘mostly patient engaged‐middling care’ (25%, *n* = 63) was characterised by moderate patient engagement with lower caregiver contribution (Figure 1 and Table S2). In this class, patients had a median age of 72 years, were almost equally distributed between males and females, had a high level of education in 40% of the cases, and low income in more than half of the cases. Furthermore, their median time since diabetes diagnosis was 12 years and almost half of them had at least one diabetes complication. Caregivers belonging to this class had a median age of 66 years, were mostly female, retired or homemakers, and had a high level of education in almost half of the cases. They had been engaged in caregiving for a median of 11 years and referred to spend a median of 3 h/day on caregiving activities. The vast majority of the dyads of this class were cohabitant and the most frequent type of relationship existing between the two members of the dyad was spousal. Compared to the other classes, patients had the highest level of education and an intermediate frequency of hospitalisations in the last year, while caregivers had at least one chronic disease more often than in the previous class (Table 3).

The third class labelled as ‘equally engaged‐high care’ (61%, *n* = 154) was characterised by the highest dyadic averages with small dyadic incongruences in type 2 diabetes care engagement (Figure 1 and Table S2). In this dyadic class, patients had a median age of 73 years, were slightly more often male than female, mostly had a low education and had a low income in more than half of the cases. Furthermore, their median time since diagnosis of diabetes was 12 years and less than 40% of them had at least one diabetes complication. Caregivers belonging to this dyadic class had a median age of 64 years, were mostly female, retired or homemakers, with a high education level in 40% of the cases. They had been engaged in caregiving for a median of 10 years and referred to spend a median of 3 h/day on caregiving activities. As in the other classes, the vast majority of the dyads of this class were cohabitant and the most frequent type of relationship existing between the two members of the dyad is the spousal one, even if in almost 30% of cases the caregiver is the adult child. Compared to the other classes, patients had the intermediate level of education and the lowest frequency of hospitalisations in the last year, while caregivers had at least one chronic disease with the same frequency as the previous class and higher than the first class. Further descriptive characteristics of the identified classes are presented in Table 3.

According to the multinomial regression model, dyads belonging to the ‘mostly patient engaged‐middling care’ class, compared to dyads belonging to the ‘equally engaged‐low care’ class, were most often characterised by patients being female (OR male = 0.12; 95% CI: 0.03; 0.51), having a higher level of education (OR = 5.64; 95% CI: 1.59; 20.00), and higher self‐care self‐efficacy (OR = 1.03; 95% CI: 1.01; 1.05), and caregivers having at least one chronic disease (OR = 4.95; 95% CI: 1.59; 15.44). On the other hand, dyads belonging to the ‘equally engaged‐high care’ class, compared to dyads belonging to the ‘equally engaged‐low care’ class were most often characterised by patients having higher self‐care self‐efficacy (OR = 1.02; 95% CI: 1.01; 1.04) and caregivers having at least one chronic disease (OR = 4.76; 95% CI: 1.73; 13.13) and higher burden (OR = 1.05; 95% CI: 1.01; 1.09) (Table 4).

**TABLE 4:** Adjusted characteristics of classes of dyadic engagement in type 2 diabetes mellitus care by multinomial regression model (number of included dyads = 212).

Characteristics	Mostly patient engaged‐middling care vs. Equally engaged‐low care			Equally engaged‐high care vs. Equally engaged‐low care		
	*β*	OR (95% CI)	*p*	*β*	OR (95% CI)	*p*
Patient						
Age (years)	−0.02	0.98 (0.92; 1.04)	0.486	−0.01	0.99 (0.94; 1.04)	0.739
Biological sex: Male vs. female	−2.12	0.12 (0.03; 0.51)	0.004	−1.06	0.35 (0.10; 1.16)	0.085
Low income: Yes vs. no	0.99	2.69 (0.94; 7.75)	0.066	0.81	2.25 (0.88; 5.78)	0.090
Marital status: Married/cohabitant vs. not married/not cohabitant	0.80	2.23 (0.50; 9.89)	0.296	−0.16	0.85 (0.23; 3.14)	0.812
Education: High school/university vs. primary/secondary school	1.73	5.64 (1.59; 20.00)	0.007	0.30	1.35 (0.41; 4.49)	0.625
Education on diabetes: Yes vs. no	−1.49	0.23 (0.06; 0.81)	0.023	−0.36	0.70 (0.24; 1.99)	0.497
Self‐care self‐efficacy (SCODI)	0.03	1.03 (1.01; 1.05)	0.006	0.02	1.02 (1.01; 1.04)	0.011
Caregiver						
Age (years)	0.00	1.00 (0.94; 1.07)	0.892	−0.02	0.98 (0.93; 1.03)	0.423
Biological sex: Male vs. female	−1.46	0.23 (0.05; 1.09)	0.064	−0.48	0.62 (0.17; 2.22)	0.465
Employment status: Employed vs. unemployed	1.33	3.78 (0.77; 18.48)	0.100	0.38	1.46 (0.36; 6.08)	0.595
Caregiving hours per day	0.03	1.03 (0.96; 1.11)	0.388	−0.04	0.96 (0.90; 1.03)	0.270
Education on diabetes: Yes vs. no	1.72	5.58 (0.99; 31.69)	0.051	−0.32	0.73 (0.15; 3.51)	0.693
Chronic disease(s): Yes vs. no	1.60	4.95 (1.59; 15.44)	0.006	1.56	4.76 (1.73; 13.13)	0.003
Quality of life (EQ5D – VAS)	0.01	1.01 (0.97; 1.04)	0.718	−0.02	0.98 (0.95; 1.01)	0.149
Burden (CBI)	0.03	1.03 (0.99; 1.08)	0.170	0.05	1.05 (1.01; 1.09)	0.023
Preparedness (CPS)	−0.06	0.94 (0.87; 1.01)	0.114	−0.04	0.96 (0.90; 1.03)	0.255

*Note:* Reference category: equally‐engaged‐low care.

Abbreviations: CBI, Caregiver Burden Inventory (0–100); CPS, Caregiver Preparedness Scale (0–32); EQ5D‐VAS, EuroQol Five‐Dimension – Visual Analogue Scale (0–100); SCODI, Self‐Care of Diabetes Inventory (0–100).

According to the multivariable linear regression model, there was an association between the class and the glycemic control measured by glycated haemoglobin. Net of the patient age, biological sex, body mass index, diabetes complications, time since diagnosis and insulin therapy, the membership in the ‘mostly patient engaged‐middling care’ class compared to the ‘equally engaged‐low care’ class, was associated with a decrease in glycated haemoglobin of (−)0.57% (95% CI: −1.07; −0.08%, Table S3). Instead, the class ‘equally engaged‐high care’, was associated with a decrease in glycated haemoglobin of (−)0.45% (95% CI: −0.89; −0.01%) as compared to the ‘equally engaged‐low care’ class. The male patient biological sex and the presence of insulin therapy were also associated with a decrease in glycated haemoglobin (*β* = −0.39: 95% CI: −0.69; −0.09 and *β* = 0.62; 95% CI: 0.29; 0.95, respectively, Table S3).

## 6 Discussion

The aim of this study was to identify and characterise distinct patterns of dyadic engagement in type 2 diabetes care. Three well characterised classes were found: ‘equally engaged‐low care’, ‘mostly patient engaged‐middling care’, and ‘equally engaged‐high care’. Several characteristics at both patient and caregiver level showed to be associated with the membership in each class. Furthermore, these dyadic classes were associated with patient glycemic control. In particular, the membership in the classes ‘mostly patient engaged‐middling care’ and ‘equally engaged‐high care’ was associated with lower levels of glycated haemoglobin compared to the class ‘equally engaged‐low care’. To the best of our knowledge, a dyadic approach accounting for the interdependence between patient self‐care and caregiver contribution was never adopted before in type 2 diabetes. Furthermore, no previous studies identified patterns of patient‐caregiver engagement in type 2 diabetes care behaviours. The availability of these patterns and their characteristics may offer a more comprehensive understanding of the type 2 diabetes care process and may represent a relevant starting point for interventions tailored to the dyads rather than individuals.

The ‘equally engaged‐low care’ class clustered dyads reporting poor execution of self‐care behaviours both in patients and caregivers, denoting minimal dyadic contribution to diabetes management by dyad members. In particular, self‐care monitoring and management behaviours were lower than self‐care maintenance behaviours. This result suggests that these dyads were quite aware of lifestyles to lead or recommend, but they seemed to pay less attention to the disease itself, both as symptoms occurrence and as consequent actions. Indeed, symptoms of type 2 diabetes may be mild and difficult to recognise and, consequently, to manage (International Diabetes Federation 2025). Nevertheless, self‐care monitoring and self‐care management behaviours are essential in the self‐care process (Riegel et al. 2012) and they had shown to be associated with patient outcomes (Fabrizi et al. 2020). Interestingly, this result is confirmed at dyadic level, as in this dyadic class, patients had the poorest glycemic control and were the most hospitalised. These dyads could be the primary focus of interventions by healthcare providers, as the empowerment of their engagement in self‐care behaviours could lead to improved patient outcomes.

The ‘mostly patient engaged‐middling care’ class clustered dyads with intermediate implementation of self‐care behaviours, with a predominant involvement of the patient over that of the caregiver. In this class, the type 2 diabetes management was predominantly achieved by the patient, while the caregiver seemed to intervene only in support or integration of the patient for the strictly necessary. Indeed, patients belonging to this class had the highest and caregiver the lowest score in self‐care maintenance behaviours, while for the other scales, their score was included between those of the other two classes for both the dyad members. Interestingly, patients belonging to this class, with respect to the previous class, were more often women and had more often a high level of education. These characteristics could support the division of tasks highlighted by the class as a consequence of the patients' capacity for autonomy, consistently with previous research (Ausili et al. 2018; Caruso et al. 2020). Furthermore, caregivers belonging to this class had at least one chronic disease more often than caregivers belonging to the previous class. Consequently, being the patient able to perform most of the self‐care behaviours autonomously, caregivers could have afforded to make a limited contribution and to be dedicated to their own condition. About glycemic control, the membership in this class showed the greatest decrease in glycated haemoglobin with respect to the ‘equally engaged‐low care’ class. This result suggests that the predominant role of the patient accompanied by a vigilant compensatory role of the caregiver in the strictly necessary areas could bring optimal benefits on patient glycemic control in the management of type 2 diabetes. Thus, patient empowerment and caregiver education in terms of support and compensation could be the most appropriate interventions for this dyadic class.

The ‘equally engaged‐high care’ class, clustered most of the dyads. Both dyad members showed higher scores than the others in almost all scales of self‐care behaviours, indicating a close collaboration between patient and caregiver in all aspects of diabetes care. With respect to the ‘equally engaged‐low care’ class, dyads belonging to this class did not have characteristics that significantly distinguished them, except for higher caregiver burden, reflecting greater caregiver involvement, and the presence of at least one chronic disease in the caregiver. This last characteristic could have led to a greater awareness of the chronic situation from the caregiver. Furthermore, the presence of chronic disease could have created different functioning conditions for the dyad with respect to the ‘mostly patient engaged‐middling care’ class. Although also in this case patients reported high self‐care scores, caregivers seemed to be involved in a virtuous circle of close collaboration with them, participating in all the activities in which the patient is already engaged (De Maria et al. 2023). This difference compared to the previous class could be traced back at least in part to the Caregiver Contribution to Self‐Care of Diabetes Inventory wording (Lee et al. 2015). In fact, the scores account for the possibility that the caregiver contribution may involve recommendation, support or even replacement for the patient. These different modes of caregiver contribution may have partially emerged in a latent form from the distinction between the ‘mostly patient engaged‐middling care’ class and the ‘equally engaged‐high care’ class identified through latent class analysis. The patient glycemic control benefited from the dyadic engagement scores in type 2 diabetes of this class, compared to the ‘equally engaged‐low care’ class. This result suggested that, even in the absence of substantial differences between groups, a greater commitment to jointly perform self‐care behaviours by patients and caregivers could be associated with lower levels of glycated haemoglobin. In these dyads could be useful to monitor the persistence of adequate scores over time for both members of the dyad, also paying attention that the burden associated with caregiving does not compromise caregivers' health and well‐being (De Maria et al. 2023).

### 6.1 Limitations

Acknowledging the study limitations, it is essential to highlight three main aspects. Firstly, the study utilised a convenience sample, which may introduce potential biases, and the sample size was quite limited. Although the available sample (251 dyads) was empirically adequate for the latent class analysis conducted, a larger sample could have provided greater statistical power and more precise estimation of class membership probabilities. Secondly, the study was conducted in a single country, so differences in results may be found elsewhere due to cultural and socioeconomic factors. Therefore, the findings of this study should be cross validated by conducting international studies. Thirdly, the cross‐sectional nature of the study did not provide information about the trend and stability over time of the results of this study.

However, the study employed valid and reliable tools to collect data. Furthermore, a dyadic approach was adopted, encompassing both patients and caregivers and providing a comprehensive understanding of the dynamics involved in type 2 diabetes care. Lastly, the integrated use of multilevel modelling and latent class analysis provided robustness in dyadic patterns identification.

### 6.2 Recommendations for Further Research

To build upon the findings of this study, future research should adopt longitudinal designs to investigate the stability and evolution of dyadic engagement patterns over time, as well as their long‐term impact on clinical outcomes such as glycemic control and hospitalisation rates. Experimental studies are warranted to evaluate the effectiveness of interventions tailored to specific dyadic profiles. Furthermore, conducting cross‐cultural and international research would enhance the generalizability of results and allow for the exploration of sociocultural and healthcare system factors that may influence dyadic dynamics in chronic illness management.

### 6.3 Implications for Policy and Practice

The identification of distinct patterns of dyadic engagement in type 2 diabetes care suggests the need for a shift towards more inclusive models of care that actively involve both patients and caregivers. Healthcare professionals should implement routine assessments of dyadic functioning during diabetes follow‐ups, employing validated instruments to evaluate patient self‐care behaviours alongside caregiver contributions. Findings from these assessments could inform individualised care plans, such as assigning tailored self‐care responsibilities, offering targeted caregiver education, or organising joint patient–caregiver training sessions to optimise dyadic engagement in diabetes care. At a policy level, these findings support the integration of dyadic approaches into chronic disease management guidelines, including explicit recommendations for caregiver assessment and involvement, funding for caregiver support programmes, and incentives for clinics to implement dyadic‐focused interventions. Collectively, these measures could facilitate caregiver involvement as a critical component of effective and sustainable care strategies and improve both patient clinical outcomes and caregiver well‐being.

## 7 Conclusion

Three distinct patterns of dyadic engagement in type 2 diabetes care were identified according to patient self‐care and caregiver contribution to it: ‘equally engaged‐low care’, ‘mostly patient engaged‐middling care’, and ‘equally engaged‐high care’. Furthermore, these classes showed differences in characteristics both at the patient and caregiver level. Lastly, an association between classes membership and glycemic control was found, integrating the pre‐existing knowledge on the relationship between patient self‐care and glycated haemoglobin.

## Author Contributions

**Diletta Fabrizi:** conceptualization, methodology, formal analysis, investigation, data curation, writing – original draft, writing – review and editing, project administration. **Paola Rebora:** conceptualization, methodology, validation, formal analysis, writing – original draft, writing – review and editing, supervision. **Maria Grazia Valsecchi:** conceptualization, methodology, validation, writing – review and editing, supervision. **Stefania Di Mauro:** conceptualization, methodology, writing – review and editing, supervision. **Michela Luciani:** conceptualization, methodology, validation, writing – original draft, writing – review and editing, supervision. **Davide Ausili:** conceptualization, methodology, validation, resources, writing – original draft, writing – review and editing, supervision, project administration.

## Funding

The authors have nothing to report.

## Disclosure

Statistical Analysis: The author team includes experienced statisticians, namely Prof. Paola Rebora and Prof. Maria Grazia Valsecchi.

## Conflicts of Interest

The authors declare no conflicts of interest.

## Supporting information


**Data S1:** Supporting Information.

**Data S2:** STROBE Statement—checklist of items that should be included in reports of observational studies.

## Data Availability

The data that support the findings of this study are available from the corresponding author upon reasonable request.
