# Supplementary material for: Dyadic Patterns of Patient and Caregiver Engagement in Type 2 Diabetes Mellitus Care: A Multicenter Observational Study
Source: J Clin Nurs. 2025 Dec 22;35(5):2342–55. doi: 10.1111/jocn.70186 (PMC13068177; doi:10.1111/jocn.70186)
Supplement: Supplementary file 1 — Data S1: Supporting Information. [file JOCN-35-2342-s002.pdf]

Supplementary Material

Article title

Dyadic patterns of patient and caregiver engagement in type 2 diabetes mellitus care: a multicenter observational study

Table 5 Performance assessment of latent class analysis models with increasing number of classes

Number of classes	Performances assessment criteria					
	BIC	< Post. prob.	< Class size	Entropy	LMR-LRT (p)	PBLRT (p)
**2**	13778.782	0.941	0.35	0.828	<0.001	<0.001
**3**	13726.389	0.910	0.14	0.871	0.019	<0.001
**4**	13692.062	0.864	0.14	0.813	0.016	<0.001

Note: Fit indices were identical when performing latent class analysis using either the coefficients from multilevel models or the observed measures as input.
Legend: BIC= Bayesian Information Criteria (the lower the better); < Post. Prob= Minor posterior probability (the average of latent class probabilities for the most likely latent class membership by latent class. It must be close to 1); <Class size= Minor class size (it must be not less than 5% of the sample); Entropy= index of model convergence (it must be close to 1); LMR-LRT (p)= p-value of the Lo-Mendell-Rubin adjusted likelihood ratio test (if p>0.05, the identified model it is not better compared to the previous one (number of classes-1)); PBLRT (p)= p-value of the parametric bootstrapped likelihood ratio test (if p>0.05, the identified model it is not better compared to the previous one (number of classes-1)(Ram & Grimm, 2009).

Figure 2 Comparison between different numbers of classes obtained by latent class analysis

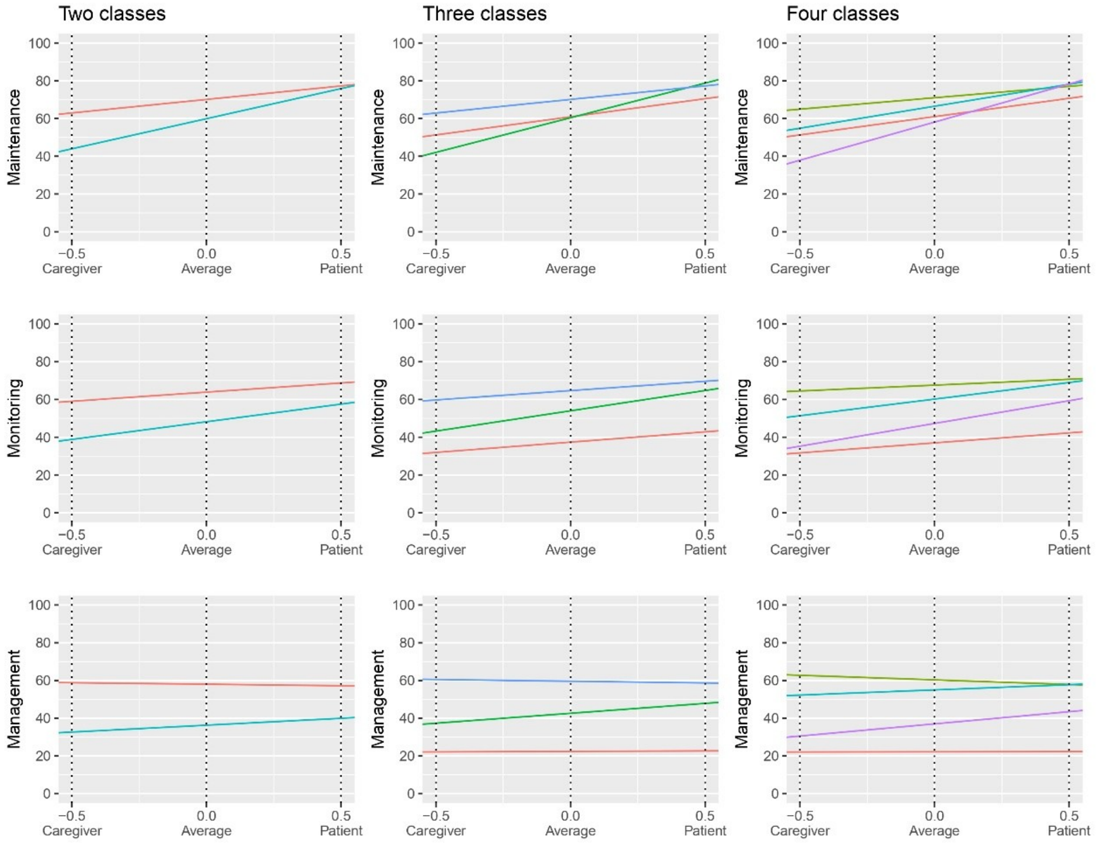

Legend: Each line coincides with one class. For each class, -0.5 on the x-axis coincides with the mean caregiver contribution to self-care maintenance, monitoring, or management score; 0.0 on the x-axis coincides with the mean dyadic engagement in self-care maintenance, monitoring, or management score. 0.5 on the x-axis coincides with the mean patient self-care maintenance, monitoring, or management score.

Table 6 Dyadic average and incongruence in self-care behaviors scales (Self-Care of Diabetes Inventory(Caregiver Contribution to Self-Care of Diabetes Inventory), as estimated by the latent class analysis in each identified pattern (n=251)

		**Equally engaged-low care**	**Mostly patient engaged-middling care**	**Equally engaged-high care**
	n (%)	34 (14)	63 (25)	154 (61)
			**Est (95% C.I.)**	
**Maintenance**	Dyadic average	60.9 (57.4; 64.4)	60.4 (58.8; 62.0)	70.1 (69.3; 70.9)
	Dyadic incongruence	19.2 (14.1; 24.3)	36.7 (33.3; 40.1)	14.4 (12.7; 16.1)
**Monitoring**	Dyadic average	37.4 (32.1; 42.7)	54.0 (50.3; 57.6)	64.7 (62.7; 66.7)
	Dyadic incongruence	10.9 (7.9; 14.0)	21.5 (19.7; 23.4)	9.9 (8.3; 11.5)
**Management**	Dyadic average	22.4 (18.9; 25.9)	42.6 (38.0; 47.2)	59.6 (56.8; 62.4)
	Dyadic incongruence	0.5 (-1.3; 2.7)	10.5 (7.8; 13.1)	-1.8 (-3.5; -0.1)

Note: The mean dyadic average and incongruence for each identified pattern were estimated by the latent class analysis, using the maximum likelihood with robust standard errors estimator.

Table 7 Association between patterns of dyadic engagement in type 2 diabetes mellitus care and glycated hemoglobin by multivariable linear regression models

	**HbA1c (%)**	**HbA1c (mmol/mol)**	
**Variable**	**β (95% C.I.)**	**β (95% C.I.)**	**p-value**
(Intercept)	7.93 (6.36; 9.49)	63.17 (46.06; 80.28)	<0.001
**Mostly patient engaged-middling care** *vs* Equally engaged-low care	**-0.57 (-1.07; -0.08)**	**-6.28 (-11.71; -0.85)**	**0.024**
**Equally engaged-high care** *vs* Equally engaged-low care	**-0.45 (-0.89; -0.01)**	**-4.88 (-9.69; -0.06)**	**0.047**
Age (years)	-0.01 (-0.02; 0.01)	-0.09 (-0.75; 0.08)	0.299
Gender: Male *vs* Female	-0.39 (-0.69; -0.09)	-4.25 (-7.53; -0.96)	0.011
Body Mass Index (kg/m^2)	0.01 (-0.02; 0.04)	0.08 (-0.22; 0.39)	0.596
Diabetes complications. Yes *vs* No	0.08 (-0.24; 0.39)	0.84 (-2.63; 4.31)	0.634
Time since diagnosis (years)	0.01 (-0.01; 0.03)	0.11 (-0.09; 0.31)	0.269
Insulin therapy: Yes *vs* No	0.62 (0.29;0.95)	6.77 (3.17; 10.38)	<0.001
